# Elucidation of flavanones, phenols and antioxidant capacity influenced by drying methods from physiologically dropped underutilized *Citrus grandis* fruits

**DOI:** 10.3389/fpls.2023.1193635

**Published:** 2023-07-10

**Authors:** Dinesh Kumar, M. S. Ladaniya, Manju Gurjar, Sachin Mendke, Sunil Kumar, Dilip Ghosh

**Affiliations:** Indian Council of Agricultural Research- Central Citrus Research Institute, Nagpur, Maharashtra, India

**Keywords:** pomelo (*Citrus grandis*), immature dropped fruits, phytochemicals, waste utilization, nutraceutical source, freeze drying

## Abstract

**Introduction:**

Nutritional content in citrus fruit is enormous. *Citrus grandis* (L.) Osbeck is underutilised citrus crop that receives little attention due to the lack of knowledge regarding its nutritional value. Citrus waste disposal poses a problem due to economic and environmental factors.

**Methods:**

The metabolites flavonoids, phenols and antioxidant capacity in the dropped fruits of the underutilised citrus species pomelo (*Citrus grandis* (L.) Osbeck) were examined.

**Results and discussion:**

Hesperidin varied from 1.22 to 2.83% and 1.08 to 1.16% from 10 mm to 14 mm whereas naringin dominates in fruits measuring 10 mm and 12mm with 60.61%, 60.77%, and 47.76%, 45.87% in freeze dried (FD) and hot air oven dried (HAOD) samples. According to the results of the antioxidant assays, the highest concentrations of ABTS azino-bis (3-ethylbenzthiazoline-6-sulfonic acid) and DPPH (2, 2-diphenyl-1-picrylhydrazyl radical) were found in freeze dried samples, ranging from 9.679 to 10.416 mmol L^-1^ Trolox and 14.825 to 16.432 mmol L^-1^ Trolox, respectively. However, the Ferric Reducing Antioxidant Power (FRAP) assay revealed higher content in samples of both FD and HAOD that were 10mm in size (4.578 mmol L^-1^ Trolox and 3.730 mmol L^-1^ Trolox). Total phenol content was measured, and the highest concentrations were found in fruits with a diameter between 10 mm and 18 mm. It ranged from 48.479 to 54.498 mg GAE L^-1^ in FD samples and from 45.757 to 51.159 mg GAE L^-1^ in HAOD samples. The smallest fruits, or those that were still in the immature stage, had the highest content. It was found that when the immature dropped fruits were dried by HAOD, the content decreased. At p<0.01 and p<0.05, there was a significant positive correlation between the flavonoids, antioxidants, and total phenols. The results showed that the immature dropped immature fruits of lesser known underutilised citrus sp. *Citrus grandis* can act as potential source of flavonoids, total phenol concentration, and antioxidant potential. Freeze drying can be recommended to recover the most bioactive substances from physiologically dropped fruits of *Citrus grandis* for use in the pharmaceutical and nutraceutical sectors. This study will help in reducing the environmental impact caused due to citrus dropped fruits and its responsible management.

## 1 Introduction

Fruit consumption has gained increasing interest among consumers due to the existence of several bioactive and its ability to protect from diseases such as diabetes, cancer, neurodegenerative, among others (Lapuente et al., 2019). The presence of bioactive chemicals neutralises dangerous reactive oxygen species for the body, preventing oxidation of vital macromolecules like DNA, RNA, and proteins and minimizing the occurrence of diseases (Zou et al., 2016). Citrus, a member of the *Rutaceae* family and the *Aurantioideae* subfamily, is one of the most significant fruit crops. *Citrus reticulata*, *Citrus sinensis*, *Citrus limon*, *Citrus aurantium*, and *Citrus paradisi* are among the citrus species grown for commercial purposes (Turner and Burri, 2013). China, Brazil, India, Mexico and United States of America are the top countries that produce citrus fruits (Marti et al., 2009).

Citrus fruits are liked all around the world for their pleasant taste, distinctive flavour, and nutritive value. Citrus fruits composed of a large number of phytochemicals and bioactive substances, such as ascorbic acid, carotene, flavonoids, antioxidants, phenolic compounds, minerals, etc. Citrus fruits have found use in the manufacturing of numerous cosmetics, functional foods, pharmaceuticals, and nutraceutical medications due to its antioxidant, anti-inflammatory, anti-cancer, and anti-fungal properties (Hayat et al., 2010; Kumar et al., 2021). Due to the diverse climatic conditions, India has a vast array of citrus genetic diversity and is also the home to numerous underutilized citrus species that are still unexplored (Kumar et al., 2021).

*Citrus grandis* (L.) Osbeck sparsely cultivated and underutilised citrus crop that receives little attention due to the lack of knowledge regarding its nutritional value. Pomelo comes in white and pink colour segments and is commonly known by other common names like Pummelo, shaddock, or Chinese grapefruit. Three main categories of citrus flavonoids include flavanones, flavones, and flavonols in which flavanones can be found as aglycones or glycosides. Hesperitin, narirutin, and didymin belong to the rutinosides group, whereas naringin, neohesperidin and neoeriocitrin belong to the neohesperidosides group. Naringenin and hesperitin comes under the aglycone forms (Tripoli et al., 2007). Reactive oxygen species that are produced under stressful circumstances can lead to the oxidation of biomolecules, which can interfere with the healthy cells’ normal metabolism and operation. Oxidative stress leads to development of several diseases, including cancer, atherosclerosis, and Alzheimer’s disease. Citrus fruits contain natural antioxidants that scavenge or neutralise dangerous free radicals, lowering the risk of disease (Kumar et al., 2019; Kumar et al., 2021). Phenolic compounds are responsible for flavour and colour of the food and have many health promoting and antioxidant properties (Gasecka et al., 2020).

Immature citrus fruits that are green in colour, drop from the stem-branch or ovary-stem junction due to physiological reasons, food deficiencies, insufficient pollination, ovule dysplasia, degeneration, or changes in endogenous hormones, etc. This phenomenon is well known as physiological dropping (Sun et al., 2015). The immature fruits that have fallen to the ground due to physiological dropping are typically dumped in the field or treated as waste. If carefully explored, these rejected dropped citrus fruits can provide a low-cost and environmentally friendly platform for the formulation of nutraceuticals or value-added food supplements. The dropped fruits can also be sold in dried form. In comparison to the conventional sun-drying process, oven drying and freeze drying are more appealing due to their easy control, industrial use, availability during off-season, higher retention of nutritional value, and low temperature and pressure operation (Sun et al., 2015; Bhatta et al., 2020).

Currently, researchers have made attempts to examine the flavonoids and antioxidants in juvenile dropped fruits of commercially cultivated citrus species, but no study has been done to elucidate the nutritional content from the dropped underutilised citrus species, i.e. pomelo. Moreover, there is relatively little knowledge about how drying methods affect the phytochemical and antioxidant content. An experiment was conducted in light of the significance and health advantages of pomelo; to understand how drying procedures affect flavonoids, antioxidant capacity, and phenolic content, as well as how effective they are at producing the highest yield.

## 2 Materials and methods

### 2.1 Chemicals and reagents

Standards of flavonoid compounds, such as hesperidin, narirutin, naringin, quercitin, and naringenin (97% purity), antioxidant standard Trolox (97% purity), radical cation ABTS^+^ azino-bis [3-ethylbenzthiazoline-6-sulfonic acid], 2, 2-diphenyl-1-picrylhydrazyl radical (DPPH), 2, 4, 6-Tri (2-pyridyl)-s-triazine (TPTZ), and gallic acid were purchased from Sigma–Aldrich (Mumbai, India). Chemicals such as sodium acetate trihydrate, ammonium acetate, acetonitrile, dimethyl sulphoxide, and acetonitrile were of the HPLC grade for use in the extraction procedure and in HPLC technique (Himedia, India). Additional chemical substances and reagents utilised in the study, such as methanol, ferric chloride, folin-Ciocalteu reagent, sodium carbonate, manganese dioxide, and 37% hydrochloric acid, were of analytical grade (Himedia, India).

### 2.2 Plant materials

**Figure 1:**
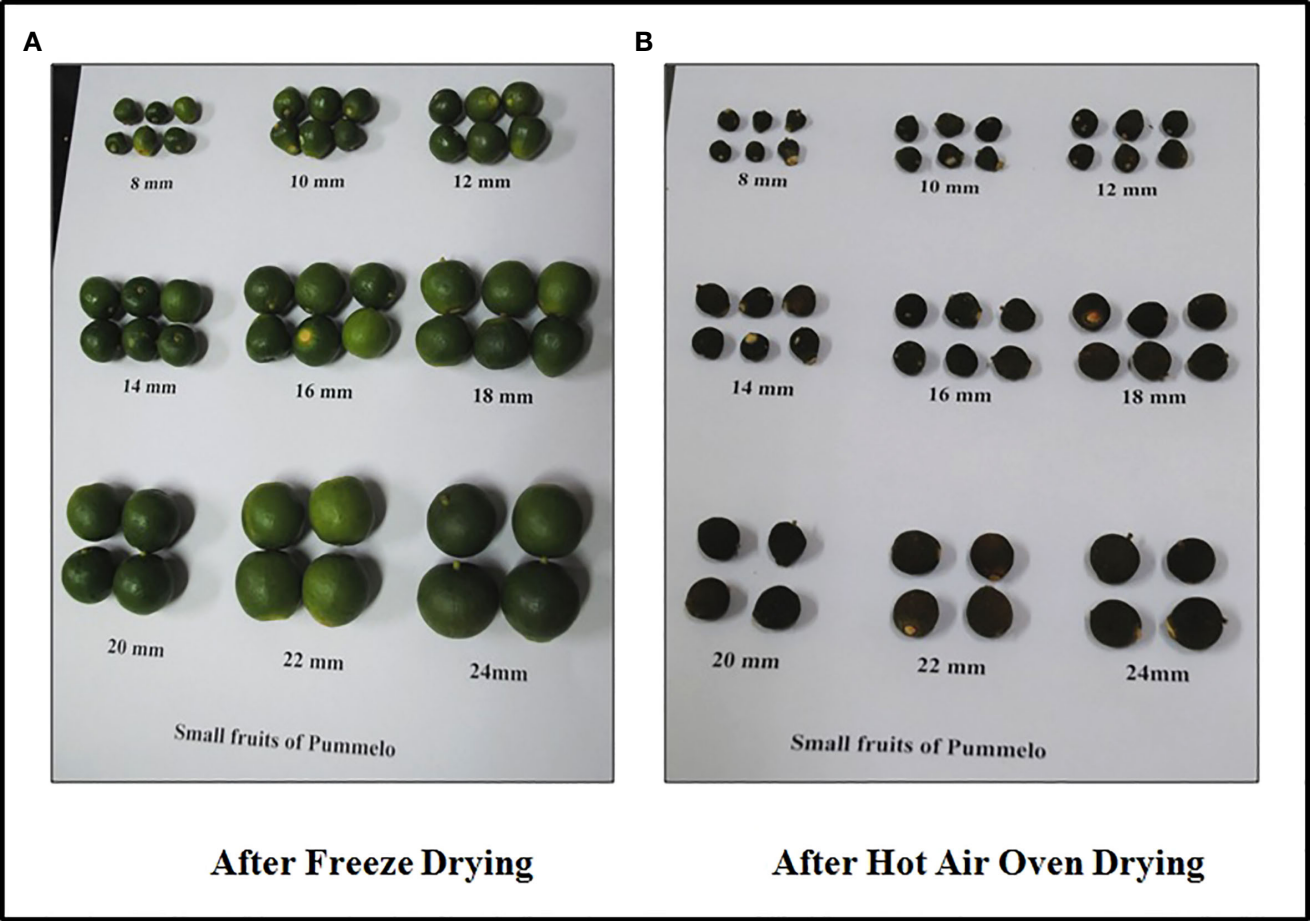
Immature Pomelo (*Citrus grandis*) dropped fruits varying from size 8 mm to 24 mm after **(A)** FD and **(B)** HAOD.

ICAR- Central Citrus Research Institute experimental blocks with the geographic coordinate’s latitude: 21°9’0”N and longitude: 79°9’0”E, respectively, were the site of collection for the immature dropped pomelo (*Citrus grandis*) fruits, which are oblate spheroid in shape (**Figure 1**). The region’s average ambient temperature at the collection time was 26.7°C, with relative humidity 60%. Its average relative humidity ranges from 13.8% to 99.3%, and its temperature ranges from 9.3°C to 43.6°C. Pummelo belongs to the *Rutaceae* family. The tree typically stands between 6 and 15 metres tall, with a 10 to 30 cm thick, crooked trunk. Spines of upto 5 cm can be found. Fruits are around 10 to 30 cm wide; the peel is clingy or more or less readily removed with greenish-yellow or pale-yellow in color. The albedo is soft, white, or pink and is divided into 11 to 18 segments and contains few, large, yellowish-white and white seeds. Samples were taken in accordance with all applicable institutional rules and regulations.

### 2.3 Drying treatment and extraction method

The collected immature dropped fruits from 8mm to 24mm were segregated and thoroughly washed with tap water in order to remove of the dirt. The samples were then sliced into 0.5 cm thickness and divided into two separate portions. One portion was stored in microwave-oven (RIVOTEK, Riviera Glass Pvt. Ltd., Mumbai, India) for 24h to 36h at 45-50°C for the hot air drying (HAOD) procedure. For the freeze drying (FD) process, a set of additional portion were placed in an ultra-low deep freezer (NEW BRUNSWICKTM, Eppendorf, India) for 12 to 24h at - 80°C, and they were lyophilized in a vacuum freeze dryer (iGene Labserve Pvt. Ltd., New Delhi, India) for 24 to 48h at − 50°C to − 55°C and at 14 to 20 Pa of pressure. The HAOD and FD samples were ground into a fine powder in a blender and passed through a sieve of 50 microns before examination. The resulting powder was kept until further study at -20°C in a deep freezer (Blue Star Ltd., Mumbai, India).

### 2.4 Determination of antioxidant capacity

Tecan Infinite M200 Pro 96-well microplate reader was used to measure antioxidant capacity (Tecan Group Ltd, Switzerland). The nitrogen radical scavenging activities i.e. ABTS and DPPH are determined as reported (Mena et al., 2011). The reaction was allowed to run at a temperature of 25°C and a wavelength of 414 nm for the ABTS assay and 515 nm for the DPPH test for 50 minutes. In both the assays, water and methanol respectively were used as blanks. Minor modifications were made to the procedure (Benzie and Strain, 1996) in order to assess the antioxidant capacity using the FRAP assay. In this assay, the FRAP reagent was prepared freshly using 300 mM acetate buffer, TPTZ solution, and ferric chloride solution in 10:1:1 ratio. A sample extract of 2 µl was then mixed with the FRAP reagent. After 40 min of reaction time at about 25°C, the absorbance was recorded at 593 nm. The results were quantified from the standard curve prepared using trolox and the results obtained were expressed as mmol L^−1^ Trolox. Three replicated trails were carried out during the experimental trials.

### 2.5 Determination of total phenol content

Total phenolic content was determined by mixing about 10 μL of sample extract with 790 μL milli-Q water, 50 μL Folin–Ciocalteu reagent and 150 μL of 20% sodium carbonate solution (Singleton and Rossi, 1965). The eppendorf tube containing the reaction mixture was shaken to agitate properly and was kept at room temp 23.5°C for 1 hr. The absorbance of the sample solution was recorded at 750 nm. The findings are reported in terms of mg GAE L^-1^ and were measured using gallic acid as the standard.

### 2.6 Determination of flavonoids composition

The Agilent Model No. 1260 Infinity System (M/s. Agilent Technologies Pvt. Ltd., United States) containing UV detector were used to analyze the flavonoid content of dropped immature pomelo fruits. Only the hesperidin and naringin flavonoids which are primarily present and persistent biomarkers in citrus fruits were found in the dropped immature fruits, despite the study carried out used the standards of flavonoids such as narirutin, hesperidin, naringin, quercetin, and naringenin respectively. The reverse phase column Nucleosil C-18 of 4.6 mm in diameter and 100 mm in length and mobile phase containing 5 mM ammonium acetate as solvent A and acetonitrile as solvent B in 75:25 (v/v) ratio was used in the analysis. The pH of the mobile phase was adjusted using acetic acid. 3 mg of the powdered sample material was mixed with 5 mL of dimethyl sulphoxide (DMSO) for extraction, and the mixture was then sonicated in a 2K1008008 series sonicator (Life-Care Equipments Pvt. Ltd., Mumbai, India). 5 μL each of sample solution as well as standard was then injected into an HPLC system for measurement after being filtered via a 0.45 µ nylon filter. The flow rate of the mobile phase was 1.0 mL/min, and the column temperature was kept at 20°C. Hesperidin and naringin, two flavonoids, were detected and quantified at 284 nm from their corresponding peak areas and calibrated against each standard (stock solution-600 ppm) diluted with the help of the mobile phase (Omidbaigi and Nasiri, 2004; Marten, 2007). The obtained results were reported as a percentage (%).

### 2.7 Statistical analysis

For each analytical parameter, three replicated measurements were conducted, and the findings were expressed as mean standard deviation (SD). For comparison and to identify significant differences in the data, Tukey’s honestly significant difference (HSD) test (multiple range test) and one-way analysis of variance (ANOVA) were performed. The correlation between flavonoids, antioxidant capacity, and total phenol concentration in the sample extracts was evaluated using Pearson correlation coefficients. The probability values (p) <0.01 was considered significantly different. After comparisons, the means in the table with the different-letter superscripts are determined to be statistically significant.

## 3 Results

### 3.1 Antioxidant capacities of dropped pomelo fruits

**Figure 2:**
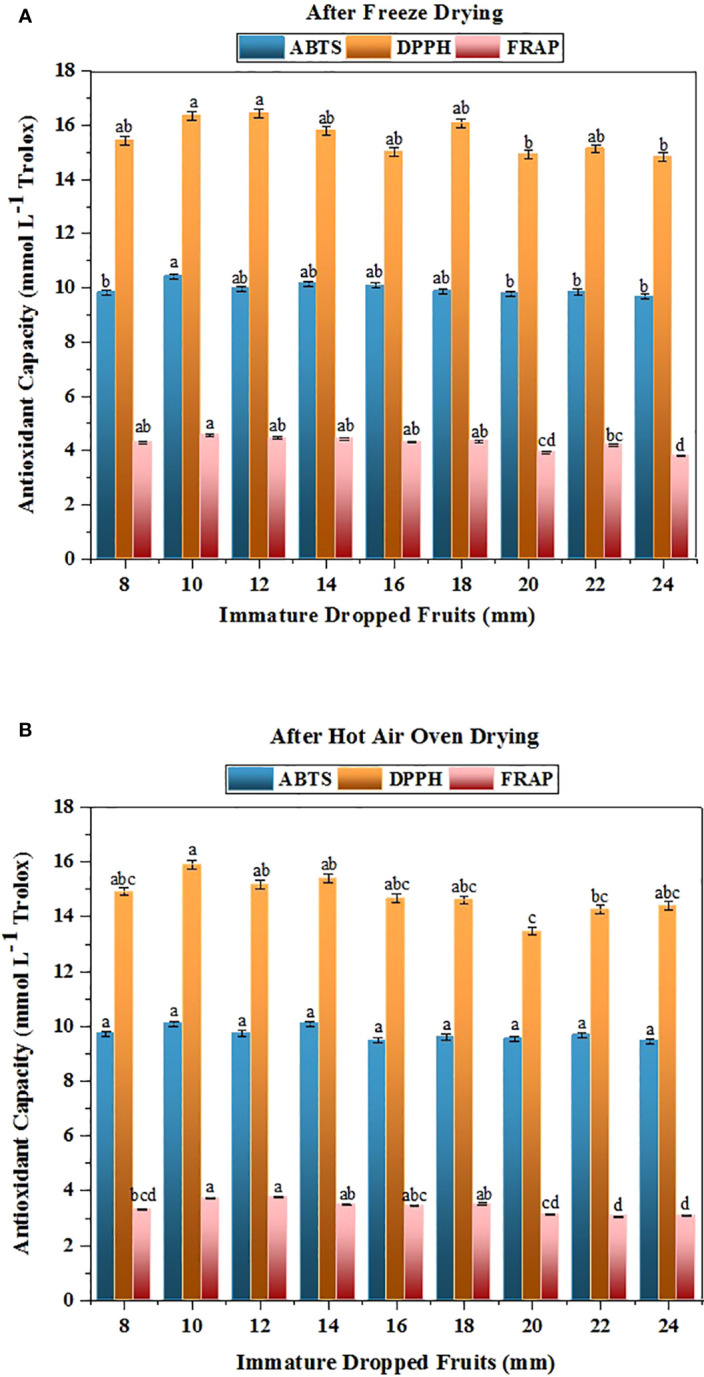
Changes in the antioxidant capacity in immature dropped fruits of Pomelo assessed by ABTS, DPPH and FRAP assay after **(A)** FD and **(B)** HAOD. The data with different superscripts are statistically significant at p<0.01 as per Tukey’s honestly significant difference (HSD) multiple range test.

Free radicals from oxygen are known for damaging the human body and leads to conditions like cancer, cardiovascular disease, and problems associated with ageing (Bellocco et al., 2009). The ABTS, DPPH, and FRAP assays were assessed to determine the antioxidant capacity. **Figure 2** illustrates the results of antioxidant capacity of dropped fruits of *Citrus grandis* that ranging from size 8mm to 24mm as measured by the ABTS and DPPH assay. When measured using the ABTS assay, the antioxidant capacity varied from 9.679 to 10.416 mmol L^-1^ trolox in FD and from 9.460 to 10.093 mmol L^-1^ trolox in HAOD samples, whereas the DPPH assay measured 14.825 to 16.432 mmol L^-1^ trolox in FD and 13.458 to 15.914 mmol L^-1^ trolox in HAOD samples. Fruit’s DPPH content decreases as it attain a higher level of maturity (Rekha et al., 2012). Lower values were obtained with the FRAP assay, which assess ferric-reducing activity, but it exhibited the same trend as the ABTS and DPPH assays (**Figure 2**). The FD samples of the pomelo dropped fruits resulted in the retention of the FRAP content values ranging from 3.803 to 4.578 mmol L^-1^ Trolox. The results from HAOD fruits ranged from 3.066 to 3.780 mmol L^-1^ Trolox. The greatest concentration was recorded in fruits of 10 mm size (4.578 mmol L^-1^ Trolox in FD and 3.730 mmol L^-1^ Trolox in HAOD). The lowest amount was quantified in dropped fruits of size 24 mm, with concentrations of 3.103 mmol L^-1^ Trolox in FD and 3.803 mmol L^-1^ Trolox in HAOD.

### 3.2 Total phenol content of dropped pomelo fruits

**Figure 3:**
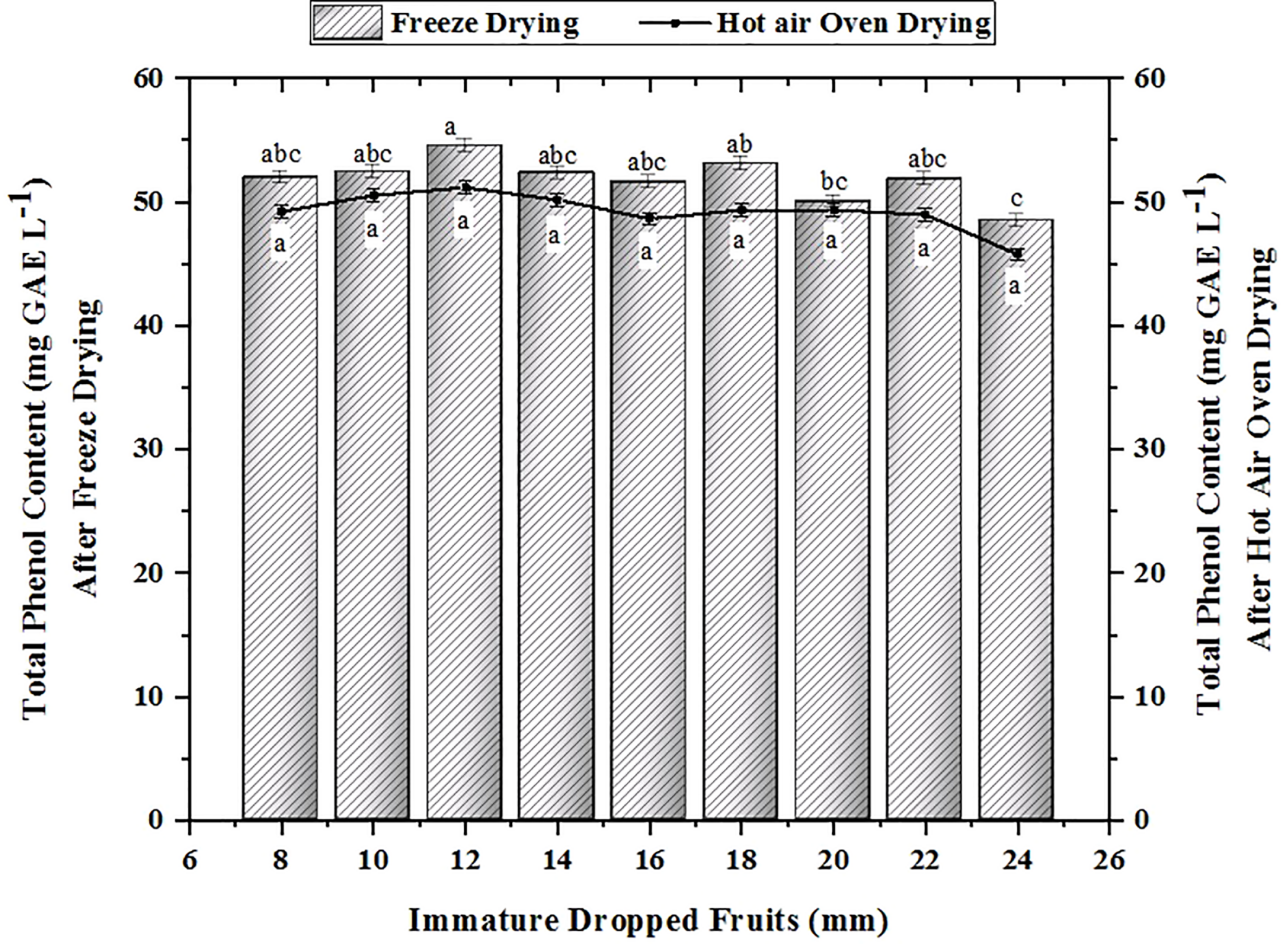
Changes in the total phenol content in immature dropped fruits of Pomelo after FD and HAOD. The data with different superscripts are statistically significant at p<0.01 as per Tukey’s honestly significant difference (HSD) multiple range test.

Phenols are regarded as one of the primary components of citrus fruits. It shields the fruit from detrimental effects of UV radiation and pathogens, as well as from predators. **Figure 3** depicts the total phenol content found in the various-sized dropped pomelo fruits. The findings indicate that the 12 mm sample had the highest total phenol content (52.403 mg GAE L^-1^ in FD and 50.530 mg GAE L^-1^ in HAOD), followed by the 18 mm and 10 mm samples, which had amounts of 53.096 mg GAE L^-1^ in FD and 49.338 mg GAE L^-1^ in HAOD and 52.403 mg GAE L^-1^ in FD and 50.530 mg GAE L^-1^ in HAOD, respectively. The findings were consistent with those of vacuum FD Citrus reticulata Blanco dropped fruits with TPC values 50.50–54.19 mg GAE L^-1^ (Kumar et al., 2021). Similarly, the concentration of total phenol decreases in Citrus sinensis L. Osbeck dropped fruits with maturity (Kumar et al., 2022a). Their findings showed that TPC content ranged from 41.736 to 55.161 mg GAE L^-1^, which was lower than our findings. The variance in the results can be attributable to different citrus species examined.

### 3.3 Flavonoid content of dropped pomelo fruits

**Figure 4:**
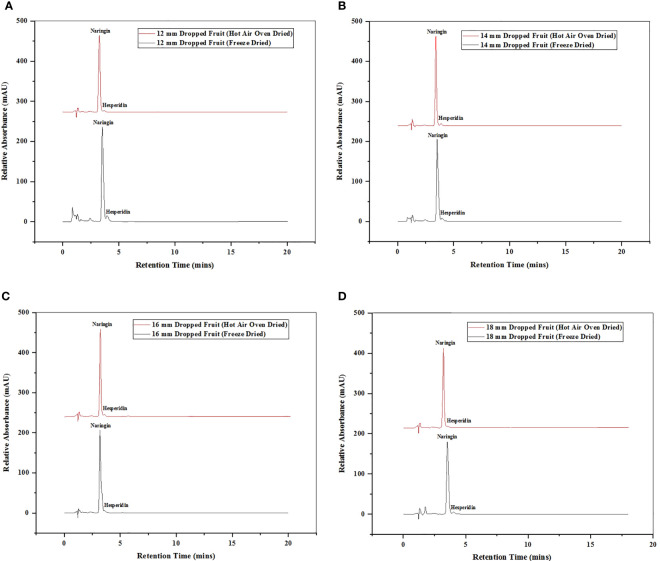
Chromatogram of HPLC of hesperidin and narinign flavonoid quantified in immature dropped fruits of Pomelo of sizes **(A)** 12 mm, **(B)** 14 mm, **(C)** 16 mm **(D)** 18 mm after FD and HAOD.

One of the main secondary metabolites, citrus flavanones, is commonly found in the diglycoside form (Tripoli et al., 2007). Statistical analysis revealed substantial significant differences in the amounts of flavonoids in the various sizes of immature dropped fruits of the pomelo (*Citrus grandis*) as shown in **Table 1**. Different fruit sizes (12 mm, 14 mm, 16 mm, and 18 mm) were compared for difference in the flavonoid compounds using HPLC chromatograms, detected at a wavelength of 284 nm (**Figure 4**). The flavonoids hesperidin and naringin were quantified with the peaks against those from standards. It was found that hesperidin has longer retention duration than naringin. Dropped fruits when assess reported naringin as the predominant flavonoid. The fruit of 10 mm, 12 mm, and 14 mm size had highest concentrations of 60.61%, 60.77%, and 55.60% in FD samples and 47.76%, 45.87%, and 45.04% in HAOD samples, respectively. When hesperidin levels were determined, they varied from 0.15 to 2.83% (FD dropped fruits) and 0.20 to 1.16% (HAOD dropped fruits). Dropped fruits ranging from 8 mm to 14 mm had the highest hesperidin content but when compared to naringin, the content was lower.

**Table 1:** Flavonoid contents (hesperidin and naringin) in immature dropped fruits of Pomelo after freeze drying and hot air oven drying.

Sr. No.	Fruit size (mm)	Drying methods	Hesperidin (%)	Naringin (%)
1	8	FD	1.210^bc^ ± 0.351	52.870^abc^ ± 2.339
		HAOD	0.930^a^ ± 0.396	42.680^abc^ ± 2.450
2	10	FD	1.220^bc^ ± 0.108	60.610^a^ ± 4.665
		HAOD	1.080^a^ ± 0.114	47.760^a^ ± 2.027
3	12	FD	2.830^a^ ± 0.173	60.770^a^ ± 1.947
		HAOD	1.160^a^ ± 0.185	45.870^a^ ± 0.830
4	14	FD	1.610^b^ ± 0.139	55.600^ab^ ± 1.057
		HAOD	0.880^ab^ ± 0.321	45.040^ab^ ± 2.933
5	16	FD	0.880^c^ ± 0.230	49.990^bcd^ ± 2.256
		HAOD	0.520^abc^ ± 0.171	41.510^abc^ ± 1.527
6	18	FD	1.230^bc^ ± 0.072	45.500^cde^ ± 1.778
		HAOD	0.470^abc^ ± 0.026	37.510^bcd^ ± 3.468
7	20	FD	0.630^cd^ ± 0.226	43.130^de^ ± 2.278
		HAOD	0.480^abc^ ± 0.122	31.060^de^ ± 2.230
8	22	FD	0.150^d^ ± 0.053	37.010^e^ ± 2.886
		HAOD	0.229^bc^ ± 0.026	35.890^cde^ ± 1.047
9	24	FD	0.760^cd^ ± 0.044	41.240^de^ ± 1.835
		HAOD	0.200^bc^ ± 0.036	29.390^e^ ± 2.629
**Tukey’s HSD at 1%**		FD	0.6389	8.8562
		HAOD	0.6951	8.001

Where, FD, Freeze drying and HAOD, Hot air oven drying.

Values are of three replicated trials ± standard deviation.

The data with different superscripts are statistically significant at p<0.01 as per Tukey’s honestly significant difference (HSD) multiple range test.

### 3.4 Correlation between antioxidants, total phenol and flavonoids

According to the parameters that were examined, the results of FD were much better than those of the HAOD approach for bioactive chemical and antioxidant capacity. The parameters of the freeze-dried samples listed in **Table 2** were correlated using Pearson’s coefficient. Citrus fruits contain larger concentrations of flavanone glycosides during the early developmental phases, or in immature fruits, than other categories of flavonoids (Omidbaigi and Nasiri, 2004; Ye et al., 2011; Lou and Ho, 2016). Naringin and hesperidin, two flavonoids, were shown to be substantially associated at p<0.01 and p<0.05 with correlation coefficients (r) of 0.804. The antioxidant tests ABTS (r= 0.716), DPPH (r= 0.764), as well as FRAP (r= 0.778) also demonstrated a statistically significantly positive correlation with naringin flavonoid at p<0.05. However, hesperidin correlated only with the DPPH with r= 0.757 at p<0.05. Hesperidin flavonoid also showed a positive correlation with TPC (r= 0.688 at p<0.05). Between the antioxidant capacity measured by the ABTS and DPPH assays and that of the FRAP assay, the correlation coefficient (r) in dropped pomelo fruits was 0.805 and 0.832, which were deemed significant at p<0.01 and p<0.05. At both p<0.01 and p<0.05, the total phenol showed a significant correlation with both DPPH (r= 0.840) and FRAP (r= 0.875). The correlation coefficient in immature calamondin peel and pulp was 0.7911 at p<0.01 (Lou et al., 2014a).

**Table 2:** Pearson’s correlation coefficient of flavonoids, antioxidants and total phenol content in dropped pomelo fruits after FD treatment.

	Naringin	Hesperidin	ABTS	DPPH	FRAP	TPC
Naringin	–					
Hesperidin	0.804**	–				
ABTS	0.716*	0.284	–			
DPPH	0.764*	0.757*	0.594	–		
FRAP	0.778*	0.559	0.805**	0.832**	–	
TPC	0.628	0.688*	0.472	0.840**	0.875**	–

**Correlation coefficient (r) values significant at p < 0.01.

*Correlation coefficient (r) values significant at p < 0.05.

Correlation coefficient (r) values not marked with any asterisk means not significant.

## 4 Discussion

In the ABTS assay, the radical cation 2, 2’-azino-bis (3-ethylbenzothiazoline-6-sulfonic acid) (ABTS•^+^) can be found as an antioxidant. Higher quenching ability shows the sample’s higher antioxidant capability (Barecca et al., 2011; Kumar et al., 2021; Kumar et al., 2022b). In the assay of DPPH, the DPPH is stable free radical. In this assay, the purple DPPH solution transforms into a colourless product indicating the presence of antioxidants. An increase level of discolouration indicates a greater antioxidant capacity (Almeida et al., 2011). The DPPH assay is used to measure both hydrophilic and lipophillic antioxidants, whereas ABTS assay reveals only the hydrophobic antioxidants (Floegel et al., 2011). The observation reveals two important features i.e. (i) the FD treatment were found to retain antioxidants more effectively than the HAOD method and (ii) the immature fruits were found to have greater antioxidant capacity in comparison to mature fruits. According to the ABTS assay, dropped fruits between the sizes of 10 mm and 16 mm had higher antioxidant capacities in both FD and HAOD fruits than mature fruits between the sizes of 18 mm and 24 mm, with a p-value of 0.01. After FD, sour cherries had a higher ABTS scavenging activity than with the other convective procedures that were used in the study (Wojdylo et al., 2013). Moreover, immature kumquats showed increased radical scavenging activity (Lou and Ho, 2017). Similar to this, fruits with a diameter between 10 and 14 mm and dropped fruits with a diameter of 18 mm had greater DPPH concentration. According to research, the juice of Chinotto fruits reported higher DPPH radical scavenging activity in immature fruits than in mature fruit juice (Barecca et al., 2010). A similar pattern was seen in the extract of thinned immature *Citrus unshiu* fruits (Kim and Kim, 2017). The ABTS and DPPH assay results are consistent with those of other researchers (Gasecka et al., 2020; Kumar et al., 2021; Kumar et al., 2022a), who also discovered a higher antioxidant content when freeze-drying the *Leccinum scabrum* (Bull.) Gray and *Hericium erinaceous* (Bull.); citrus fruits namely *Citrus reticulata* Blanco and *Citrus sinensis* L. Osbeck respectively. The findings of FRAP assay pointed out a direct relationship between the content and the drying method used. Similar results were found while studying the effect of drying process on Nagpur mandarin (Kumar et al., 2021), immature mandarin fruits (Ye et al., 2011), and physiological drop citrus fruits (Sun et al., 2013; Kumar et al., 2021), respectively. In contradiction to the results obtained, the FRAP activity was found to be lower in the unripe chinotto fruits (Barecca et al., 2010). Due to prolonged exposure to hot air, the HAOD method caused oxidation. On the other hand, the FD approach operated at lower air pressure for a shorter period of time and thus lessened this oxidation effect (Wojdylo et al., 2013).

The amount of total phenolic content was greatly impacted by the drying process. The largest phenolic concentration was found in FD samples operated at temperatures between −50°C to −55°C for 24 to 48 h compared to samples dried in hot air ovens at 45-50°C for 24 to 36 h, which had a lower phenolic content. The primary enzyme in the phenylpropanoid pathway for the biosynthesis of phenolic compounds is phenylalanine ammonia lyase. The decrease in the activity of this enzyme during the citrus fruit developmental stages and simultaneous increase in the activity of polyphenol oxidase enzyme is attributed to the decrease in the content of total phenols (Gupta et al., 2021).The varying concentration could also be due to the higher temperature of hot air oven (Wojdylo et al., 2013). The findings showed that the content of the dropped immature fruits get influenced during their maturation stages. Phenolic compounds acts as antioxidants (Rice-Evans and Miller, 1996). As a result, samples with greater phenol contents had higher antioxidant potential (Buyukkormaz and Kucukbay, 2022). Similar observations were seen with vacuum-drying of kumquats (Ozcan-Sinir et al., 2019). The findings are consistent with the research done using rose hip (Rosa rubiginosa) and persimmon leathers (Karaman et al., 2014; Ruiz et al., 2014). FD samples when assessed recorded minimum degradation of phenolic content. Several researchers observed similar results when drying sour cherries (Wojdylo et al., 2013), kumquat (Lou et al., 2015); studying maturity stages with Citrus unshiu (Kim et al., 2022), Citrus aurantium (Mansour, 2018) and thinned immature Citrus unshiu (Kim and Kim, 2017) respectively. On the other hand, hot air drying enhanced the total phenol content (TPC) of the aqueous extract of dried lemon (Citrus limon) pomace (Papoutsis et al., 2017). The total phenol concentration observed in our investigation is in accordance with earlier studies done on mandarins (Ye et al., 2011), immature citrus fruits, green and ripe Chinotto (Citrus x myrtifolia Raf.) fruits (Barecca et al., 2010).

Hesperidin is the main flavonoid in mandarin, sweet orange, and lemon, whereas naringin is mostly present in the citrus species of sour orange, pummelo, and grapefruit (Dhuique-mayer et al., 2005; Vanamala et al., 2006). The amount of flavonoids varies greatly amongst citrus species. The variable content is caused by a number of factors, including genetic and environmental, geographic origin, meteorological conditions, soil qualities, time of fruit collection, storage, portions of the fruit, etc (Lu et al., 2006). According to the experimental findings, drying methods and flavonoid content are interconnected. The content was found maximum in the vacuum FD samples. Immature calamondin peels, an underutilised citrus species, showed the same behaviour (Lou et al., 2014b). Due to increased PPO activity and decreased chalcone synthase gene expression, there is a drop in flavonoid concentration during citrus fruit development (Gupta et al., 2021). The flavonoid content of *Citrus unshui* decreased with increase in maturity (Kim and Lim, 2020; Kim et al., 2022). Calamondin, *Citrus grandis* Osbeck, Chinotto (*Citrus myrtifolia* Raf.), and other immature citrus fruit extracts likewise showed a shifting pattern in flavonoid content (Barecca et al., 2010; Lou et al., 2014b; Kim and Kim, 2017). The study’s findings were consistent with those of experimental tests done on grape skin and immature physiologically dropped citrus fruits (Sun et al., 2013; Sun et al., 2015; Kumar et al., 2021; Kumar et al., 2022a). Finally, it can be said with certainty that the FD approach should be used to retain the flavanone glycosides hesperidin and naringin.

Significant correlation was observed between antioxidants, total phenol and flavonoids as per the results obtained. The study with *C. aurantium* citrus fruits found similar results with significant correlation (Mansour, 2018). Flavonoids are responsible for antioxidant capacity (Sun et al., 2013). Other researchers have also found positive correlation between total phenol and antioxidant compounds in Satsuma mandarin and Ponkan, immature kumquat and *Citrus sinensis L.* Osbeck fruits (Xu et al., 2008; Lou et al., 2015; Kumar et al., 2022a). Phenolic molecules are thought to contribute significantly to antioxidant capability (Xu et al., 2008). More is the total phenol content; more is the antioxidant capacity (Rice-Evans and Miller, 1996). The findings are consistent with research undertaken with physiological drops in citrus fruits, respectively (Sun et al., 2013; Kumar et al., 2021).

The findings of the current study will increase consumption of the little-known, underutilised citrus fruit pomelo in light of the growing consumer interest in items with authentic nutritional content. At the same time, the study will encourage industrial applications of dropped fruits towards nutraceutical formulations, herbals, etc. The study will also address the issue of citrus dropped fruits as waste and will contribute in valorization. The socioeconomic status of the citrus growing region will also improve as a result of the study.

## 5 Conclusions

In this paper, we investigated the bioactive components, primarily flavonoids, antioxidants, and total phenol of immature dropped fruits of underutilised pomelo species and determined their correlation. Furthermore, we studied the impact of FD and HAOD on the assessed components. We found that Naringin was the main flavanone glycoside present in all the different sized fruits with the highest level in the 10 mm and 12 mm sizes. Next, in comparison to other dropped fruits, immature fruits with sizes ranging from 10 mm to 16 mm were found to have abundant antioxidant capacity measured by ABTS, DPPH, and FRAP assay. In case of total phenol content, fruits between 20 and 24 mm in diameter had the lowest levels and those between 12 and 18 mm in diameter had the greatest levels. Flavonoids and total phenol contributed well to the antioxidant capacity and significant correlated at p<0.01 and 0.05. The results of the study highlights that (1) Drying effect had a substantial impact on the flavonoids, antioxidant potential, and total phenol content of dropped fruits and (2) FD performed at − 50°C to − 55°C for 24h to 48h was found to be more efficient for obtaining maximum recovery than hot air oven drying at 45-50°C for 24h to 36h. This kind of approach appeared to very useful and offer crucial information regarding the bioactive components of underutilised citrus spp. *Citrus grandis* focusing mainly the dropped fruits which are usually unexplored due to the lack of information. The FD examined reveals great potential applications which can be adapted in the citrus industry in the future.

## Data availability statement

The original contributions presented in the study are included in the article/**Supplementary Material**. Further inquiries can be directed to the corresponding author.

## Author contributions

DK conceptualized, designed and wrote the first manuscript. MG performed the experiment, supported data analysis, assisted with the manuscript’s writing and editing. ML and DG administered and supervised the research, SM performed the experiment. SK gathered the resources. All authors contributed to the article and approved the submitted version.
