# Supplementary figures and images for: Elucidation of flavanones, phenols and antioxidant capacity influenced by drying methods from physiologically dropped underutilized Citrus grandis fruits
Source: Front Plant Sci. 2023 Jul 10;14:1193635. doi: 10.3389/fpls.2023.1193635 (PMC10363982; doi:10.3389/fpls.2023.1193635)

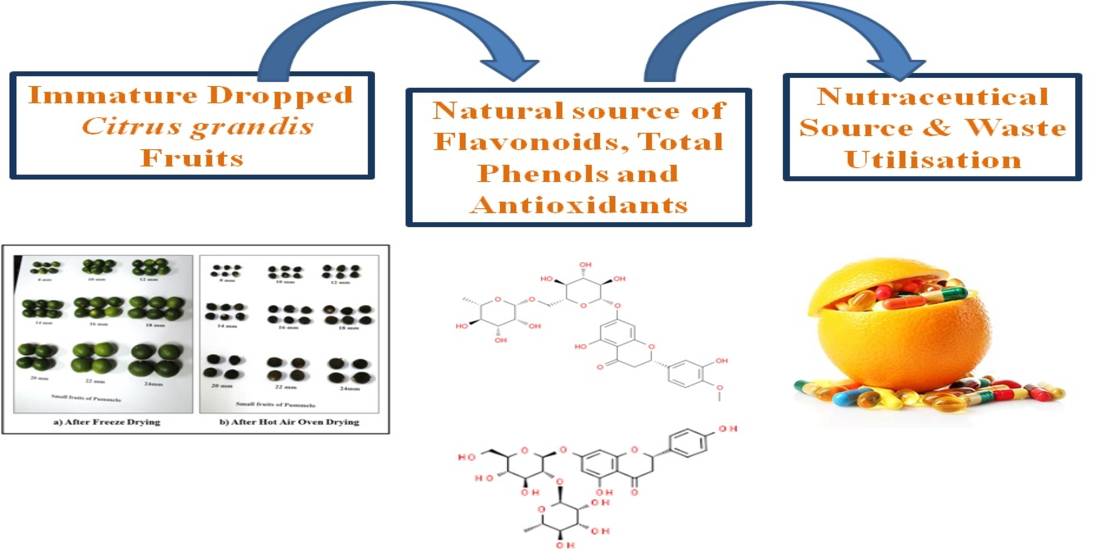

Supplement: Supplementary file 1 [file Image_1.tiff]
